# State-transition and simulation-based modeling approaches for simulating the progression of dental caries: a scoping review

**DOI:** 10.3389/froh.2026.1791208

**Published:** 2026-05-14

**Authors:** Xenia Teplitzky, Darja Gostilo, Georgios Tsilingaridis, Blend Hamza, Līna Petrova, Shaju Jacob Pulikkotil

**Affiliations:** 1 Prof. Shaju Jacob Pulikkotil Group. Oral Health Research, Faculty of Dentistry, Rīga Stradiņš University, Riga, Latvia; 2 Division of Paediatric Dentistry, Department of Dental Medicine, Karolinska Institutet, Stockholm, Sweden; 3 Center of Paediatric Oral Health Research, Stockholm, Sweden; 4 Clinic of Orthodontics and Pediatric Dentistry, Center for Dental Medicine, University of Zurich, Zurich, Switzerland; 5 “Zobu feja” dental clinic, Riga, Latvia

**Keywords:** dental caries, disease progression, Markov chains, modeling approaches, pediatric dentistry

## Abstract

**Introduction:**

Dental caries is a common chronic disease in children, influenced by biological, behavioral, and environmental factors, and modeling approaches provide a structured way to simulate disease progression, evaluate preventive or therapeutic interventions, assess economic outcomes, and inform policy. Despite increasing use of decision-analytic and simulation-based frameworks in pediatric dental caries, a comprehensive overview of model types, structures, and applications is lacking. The objective of this scoping review is to identify and describe state-transition and simulation-based modeling approaches used to simulate the progression of dental caries in children, including models applied to the evaluation of preventive or therapeutic interventions and economic outcomes, and to summarize their structural characteristics, applications, and methodological features.

**Methods:**

This review includes studies of children and adolescents (birth to 18 years) from any health status or risk profile and in any geographic or clinical setting. Eligible studies described, developed, or applied state-transition or simulation-based models of dental caries progression, while studies limited to predictive, statistical, or cross-sectional approaches, as well as reviews and commentaries, were excluded. MEDLINE (PubMed) and Scopus were searched using a structured strategy, limited to English-language publications. Studies were screened and charted key model features independently by two authors.

**Results:**

A total of 43 studies were included, most using state-transition approaches, primarily Markov cohort models and Markov microsimulations. Model structures ranged from simple caries presence to multi-state models incorporating enamel-dentin progression, ICDAS stages, or restorative pathways.

**Discussion:**

These findings highlight how structural and methodological choices in caries modeling determine the scope of addressable research questions and underscore the need for standardized health-state classifications.

**Registration:**

This review was registered in the Open Science Framework, DOI 10.17605/OSF.IO/6BT7K.

**Systematic Review Registration:**

https://archive.org/details/osf-registrations-6bt7k-v1

## 1 Introduction

Dental caries is among the most prevalent chronic conditions in children worldwide and poses a major public health issue. Despite improvements in preventive dental care, many children still develop caries, which may result in pain, infection, impaired function, and diminished quality of life. Early development of dental caries can affect the permanent teeth formation and carries long-term implications for both oral and general health (1–3). Caries progression involves repeated cycles of demineralization and remineralization, with lesions potentially advancing from early enamel changes to cavitated dentin lesions and ultimately tooth loss if untreated (4).

This review adopts a methodological interpretive lens, treating each included study as a case study in modeling design decisions, rather than evaluating the clinical effectiveness of specific interventions. Modeling approaches describe transitions between health states, capture disease progression over time, and assess the effects of preventive or therapeutic interventions (5). They are also widely used in health economic evaluations to estimate long-term costs and outcomes of interventions and to inform policy and resource allocation decisions (6). By synthesizing evidence from clinical trials, observational studies, and real-world data, models can project outcomes beyond empirical follow-up, generating estimates of long-term disease burden, effectiveness, and uncertainty (5). In pediatric dental caries, modeling approaches are particularly important due to the disease's chronic, progressive nature and multifactorial etiology, which short-term studies cannot adequately capture.

Modeling frameworks differ in complexity, assumptions, and scope—from simplified state-transition structures in Markov-based modeling frameworks to detailed representations of individual behavior in agent-based modeling frameworks (7–10)—influencing their suitability depending on disease, data, and research aims. Unlike statistical prediction or machine learning models, which estimate risks at specific time points, simulation-based modeling approaches explicitly represent transitions between disease states over time. This feature is particularly relevant for dental caries, a chronic condition characterized by gradual progression through clinically distinguishable stages and by repeated opportunities for prevention and treatment. Simulation-based frameworks allow researchers to represent these dynamic processes, evaluate alternative intervention strategies, and project long-term outcomes beyond observed study periods. For this reason, the present review focuses specifically on simulation-based modeling approaches that explicitly represent disease progression.

This scoping review aims to identify and describe the modeling approaches used to simulate dental caries progression in children, including models developed to evaluate preventive or therapeutic interventions and economic outcomes, and to summarize their structural features, applications, and methodological characteristics. By presenting an organized overview of existing models, this review intends to support researchers in making informed methodological choices and to encourage greater transparency in future modeling work.

Specifically, it answers the following research question: What modeling approaches are used to simulate the progression of dental caries in children, and what are their key characteristics and applications, including intervention and economic evaluation contexts?

## 2 Methods

This scoping review was conducted in accordance with the JBI methodology for scoping reviews (11) and reported in line with the Preferred Reporting Items for Systematic Reviews and Meta-Analyses extension for Scoping Reviews (PRISMA-ScR) (12). This review was conducted in accordance with an *a priori* protocol (13) which is part of the registration on OSF. This review has been registered in https://osf.io/6bt7k/.

### 2.1 Conceptual background and rationale

#### 2.1.1 Conceptual framing

In this review, terminology distinguishes models (specific implemented representations within a study), modeling frameworks (broader classes such as Markov or microsimulation models), and modeling approaches (umbrella term for referring collectively to these methods). Included models are interpreted through a methodological lens, focusing on structural design decisions and their implications for research applicability. This review considers structural complexity (number and definition of health states, handling of progression and reversibility), temporal resolution, and decision relevance (e.g., policy evaluation, burden estimation, or methodological exploration). This framing allows comparison across studies despite differing research questions or data availability.

Although multiple individual studies have used modeling approaches to examine dental caries, the overall landscape remains fragmented. Some studies examine the cost-effectiveness; others estimate disease burden, and some rely on statistical prediction methods rather than dynamic disease modeling approaches (14–16). Currently, no comprehensive overview exists describing the types of models, their structures, and their purposes in pediatric populations with dental caries.

Synthesizing this evidence is crucial to support researchers in selecting appropriate modeling approaches for future work and ensuring that models sufficiently represent the caries process in children.

Because the field spans both clinical and methodological disciplines, researchers may use varying terminology, levels of detail, or conceptual modeling frameworks when describing disease processes. As a result, it is not always straightforward to compare studies or understand how one modeling framework relates to another. For instance, cohort-based Markov models track groups of patients through a limited number of health states and are generally suited for population-level or policy analyses. Microsimulation-based models simulate individuals separately, capturing heterogeneity in risk, treatment history, or preventive care exposure. Surface- or tooth-level models extend these modeling frameworks to finer granularity, enabling detailed assessment of lesion progression, restorative sequences, and preventive interventions. Providing clarity through synthesis can therefore support more consistent communication across research groups and assist newcomers in navigating the available methodologies. Furthermore, compiling existing modeling approaches may help highlight areas where certain types of models are underused or where structural features have not been explored, offering insight into opportunities for methodological development.

#### 2.1.2 Key terms and operational definitions

For this review, dental caries is defined as the localized breakdown of hard dental tissues due to bacterial activity, progressing from early enamel lesions to cavitated lesions. Pediatric populations refer to individuals from birth through 18 years of age. Modeling approaches encompass formal, quantitative, or simulation-based methods describing disease progression, including decision-analytic, Markov, microsimulation, agent-based, and system dynamics modeling frameworks. Only studies simulating disease progression—i.e., transitions between caries states over time—were included; statistical prediction or risk factor analyses without progression modeling approaches were excluded.

#### 2.1.3 Preliminary search and rationale

A preliminary search of MEDLINE (PubMed), the Cochrane Database of Systematic Reviews, and JBI Evidence Synthesis was conducted. Three recent reviews on related topics were identified: Bhadila et al. performed a scoping review of recurrent caries models related to dental restorations (17), Havsed et al. systematically reviewed multivariable prediction models of caries increment (18), and Tresna et al. explored compartmental model utilization for studying the role of bacteria, mainly Streptococcus Mutans, in dental caries (19). Bhadila et al. focused on restoration outcomes, whereas Havsed et al. examined statistical prediction rather than simulation-based disease progression, and Tresna et al. focused more on the role of bacteria rather than the progression of the disease. Neither review comprehensively maps the types, structures, or purposes of modeling frameworks for pediatric caries.

Individual studies using simulation or state-transition models exist, indicating sufficient primary evidence for a scoping review. The diversity of modeling approaches across studies—including differences in time horizons, population subgroups, and model purposes—reinforces the value of a scoping review to document model types and operationalization. A scoping review enables broad mapping of characteristics and uses of modeling approaches in pediatric dental caries, identifies methodological gaps, and supports future research without restricting eligibility to certain outcomes or study designs.

### 2.2 Inclusion criteria

#### 2.2.1 Participants

This review considered sources that included children and adolescents, defined as individuals from birth up to 18 years of age. Studies may have included participants with any general health status, risk profile, or specific subpopulation, such as children at high risk of dental caries or children with special healthcare needs.

Inclusion criteria:

- Studies that model the progression of dental caries in individuals under 18 years.
- Studies that include primary data, secondary analyses, or simulation-based approaches applied to pediatric populations.

Exclusion criteria:

- Studies focused exclusively on adults (≥18 years).
- Studies that do not simulate disease progression, such as those only modeling restorative outcomes or evaluating risk factors without a progression framework.

#### 2.2.2 Concept

This review considered sources that explored modeling approaches used to simulate the progression of dental caries in children. Studies were included if they describe, develop, or apply quantitative or simulation-based models to represent how dental caries evolves over time in pediatric populations.

Included modeling approaches may have encompassed, but were not limited to:

- State-transition (Markov-based modeling frameworks)
- Microsimulation-based modeling frameworks
- Agent-based modeling frameworks
- System dynamics modeling frameworks
- Decision-analytic modeling frameworks

Studies employing statistical or computational approaches were included only if they simulated transitions between caries states over time; purely predictive, cross-sectional, or regression-based modeling approaches were excluded. This focus ensured inclusion of modeling frameworks that represent disease dynamics rather than single-timepoint risk estimation, consistent with the review's emphasis on simulation-based and state-transition modeling approaches.

Exclusion criteria:

- Studies that focus solely on treatment effectiveness, restorative outcomes, or intervention cost-effectiveness without modeling disease progression.
- Studies that are purely descriptive or cross-sectional without any modeling component.

The review extracts and summarizes key features of the models, including:

- Model type and structure
- Health states or stages represented
- Time horizon and cycle length
- Assumptions and data sources
- Applications (e.g., policy, clinical decision-making, or research).

#### 2.2.3 Context

This review considered sources that were conducted in any geographic, clinical, or community setting, without restriction. This included:

- Healthcare settings: dental clinics, hospitals, or specialized pediatric care centers.
- Community settings: schools, public health programs, or population-based cross-sectional surveys.
- Geographic scope: studies from any country, including low-, middle-, and high-income settings.

No restrictions were applied regarding:

- Socioeconomic status of participants
- Health system type
- Cultural or regional differences

Rationale: The inclusion of studies from any context ensured a comprehensive mapping of modeling approaches and allows identification of differences in model structure, application, and assumptions across diverse settings.

### 2.3 Types of sources

This scoping review considered study designs that developed, described, applied, or validated modeling frameworks simulating the progression of dental caries in children. Eligible studies may have included those employing decision-analytic, state-transition (Markov-based), microsimulation-based, agent-based, or system dynamics modeling frameworks, as well as statistical or machine learning approaches representing disease progression over time.

Systematic reviews^1^, narrative reviews, commentaries, and opinion papers were excluded.

This approach is consistent with the JBI methodology for scoping reviews, which supports the inclusion of diverse evidence sources relevant to the review question.

### 2.4 Search strategy

The search strategy aimed to locate published studies only. The search was built upon the strategy previously developed for a related systematic review on transition probabilities and disease progression in pediatric dental caries (20). The search terms and structure were adapted to identify literature that described modeling approaches to simulate the progression of dental caries in children. The search was conducted on October 29th, 2025. The full search strategies are provided in Supplementary Appendix I.

The databases searched were MEDLINE (via PubMed) and Scopus (Elsevier). These databases were selected because they provide extensive coverage of biomedical and interdisciplinary literature and are widely used sources for systematic and scoping reviews. MEDLINE provides comprehensive indexing of biomedical research, while Scopus includes a broader range of journals across health sciences, social sciences, and related fields, including many sources not indexed in MEDLINE.

Transparent and reproducible search strategies are a fundamental requirement of systematic and scoping reviews (12). While broader search platforms such as Google Scholar or Dimensions may retrieve additional records, their search algorithms are not fully transparent, and their results are difficult to reproduce systematically. Therefore, MEDLINE and Scopus were used as the primary databases for the structured search.

Reference lists of included articles were not screened for additional papers, as the database search was intentionally designed to be broad and highly sensitive, combining multiple terms for the disease (dental caries), population (children), and modeling of disease progression. Given the scope and specificity of the search strategy, it was considered unlikely that reference list screening would identify a substantial number of additional eligible studies.

Sources published in English were included due to resource constraints for translation. Non-English language was an exclusion criterion. This restriction was deemed unlikely to substantially bias the findings, as most of the relevant research on pediatric dental caries and disease progression modeling is published in English. All search results retrieved were in English. Sources published from database inception to October 29th, 2025, were included as this scoping review aimed to comprehensively map the literature on disease progression modeling in children with dental caries.

### 2.5 Study/source of evidence selection

Following the search, all identified records were collated and uploaded into Mendeley (web) (Mendeley Ltd., Elsevier, Netherlands) and duplicates were removed. Following a pilot test, titles and abstracts were screened by 2 independent reviewers for assessment (XT and DG) against the inclusion criteria for the review. Due to the relatively small number of records retrieved after deduplication (*n* = 70; see Supplementary Appendix I), title/abstract screening and full-text screening were conducted in a single step. Although this approach deviates from the standard two-stage screening process, it was deemed appropriate to increase efficiency while maintaining rigorous application of the predefined inclusion criteria by two independent reviewers (XT and DG). Provided, the reviewers were able to retrieve the full text. For all records, the title and abstract were screened first, and, if not excluded based on title and/or abstract, the full text was retrieved for screening. Details on inclusion and exclusion can be found in the Supplementary Table S1. Citation details were managed in Microsoft Excel for record-keeping and eligibility tracking. Although the protocol specified that citations would be imported into JBI System for the Unified Management, Assessment and Review of Information (JBI SUMARI) (JBI, Adelaide, Australia) (21), this was not undertaken due to lack of access to the software. To mitigate this deviation, all records (already collated and de-duplicated in Mendeley as described above) were systematically tracked in Excel, and screening and data extraction procedures were documented in detail to ensure transparency and reproducibility. While using JBI SUMARI may offer standardized functionality, the combined use of Mendeley and Excel maintained methodological rigor, and this deviation is not expected to have affected the comprehensiveness or validity of the review findings. Full-text sources that did not meet the inclusion criteria were excluded, and reasons for their exclusion are provided in Supplementary Appendix II. Any disagreements that arose between the reviewers were resolved through discussion.

### 2.6 Data extraction

Data were extracted from papers included in the scoping review by 2 independent reviewers (XT and DG) using a data extraction tool developed by the reviewers, provided in Supplementary Appendix III. The pilot data extraction is provided in the Supplementary Table S2. The data extracted included specific details about the population characteristics, the modeling approach (e.g., the type of model and time horizon), the purpose/application of the model, reported strengths and limitations and key outputs of the model relevant to the review question. Any disagreements that arose between the reviewers were resolved through discussion. It was not required to contact authors of papers.

Consistent with JBI guidance for scoping reviews, no formal critical appraisal of included studies was conducted. Scoping reviews aim to map the extent, range, and nature of research evidence rather than evaluate methodological quality. As the JBI Handbook notes, qualitative content analysis in scoping reviews is generally descriptive, and undertaking thematic analysis or other forms of synthesis would go beyond the objectives of a scoping review (11). Omitting critical appraisal is therefore methodologically appropriate, allowing for a comprehensive overview of available modeling approaches while maintaining transparency and reproducibility.

### 2.7 Data analysis and presentation

Extracted data was collated, summarized, and presented in tabular and narrative formats to map the current landscape of modeling approaches used to simulate dental caries progression in children. A descriptive analysis was performed to summarize the frequency and distribution of model types, structures, and applications across studies.

Where studies incorporated economic evaluations, economic outputs such as cost-effectiveness results, incremental cost-effectiveness ratios, and quality-adjusted outcomes were recorded as part of the key outputs field in the extraction table. No separate economic extraction framework was applied; economic parameters were captured descriptively as part of the broader characterization of model applications and purposes.

Tables and figures were used to display:

- The range of modeling approaches identified (e.g., Markov-based, microsimulation-based, or agent-based modeling frameworks).
- The characteristics of each model including structure, time horizon, data sources, and validation methods.
- The intended use or purpose of each model (e.g., disease progression, intervention evaluation, and economic assessment).

A narrative synthesis accompanies the tables to provide an interpretive overview of modeling trends, highlighting similarities and differences in methodological approaches, as well as potential gaps in current modeling practices.

## 3 Results

### 3.1 Source of evidence inclusion

**Figure 1:**
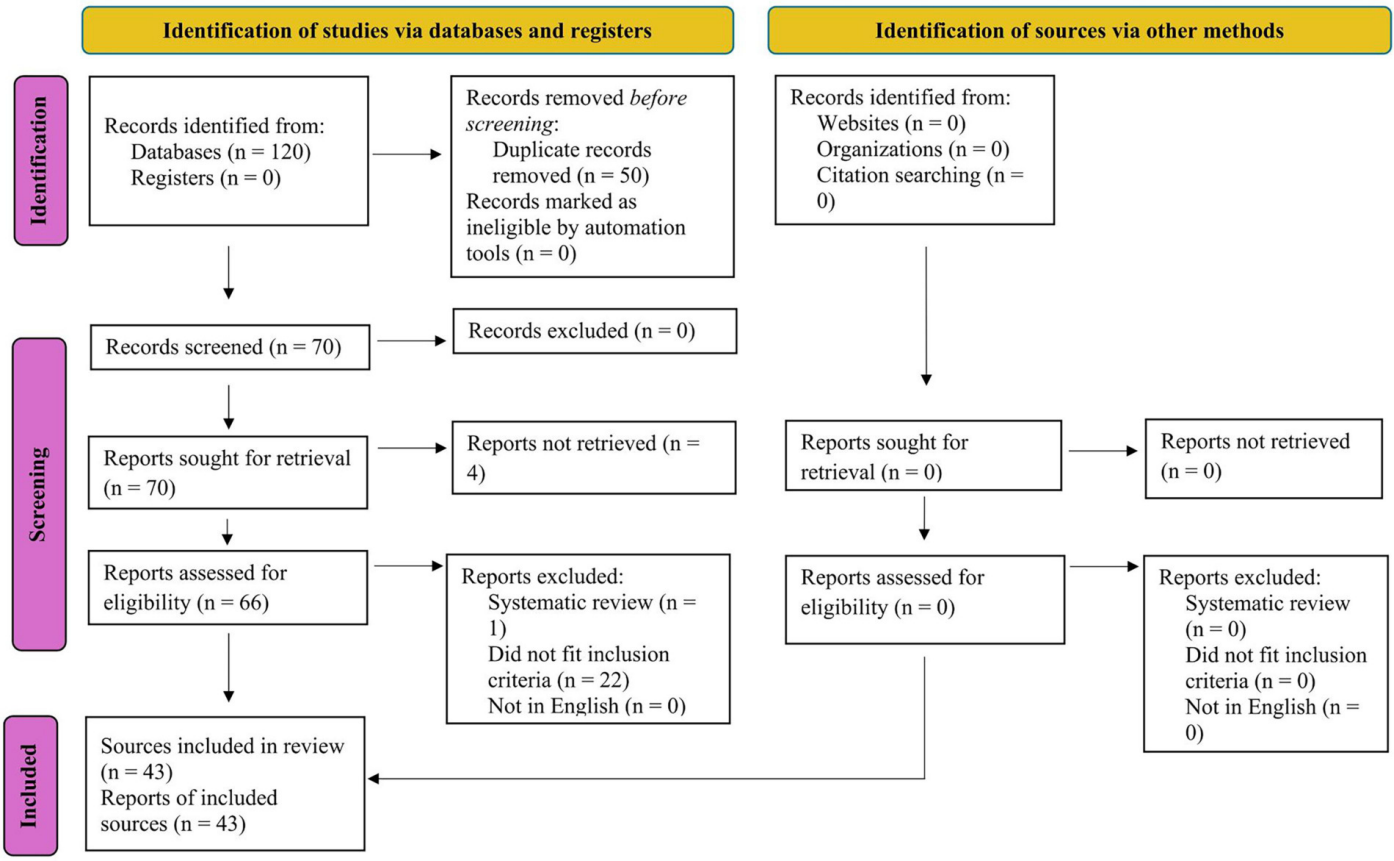
Flowchart, record inclusion. Alt text: This figure shows a flowchart that depicts how many records were found in the searches, how many were excluded and for what reasons, and how many were included in the final review.

A total of 120 records were identified through database searches. After removing duplicates (50 records), 70 records were identified for full text retrieval. Four full texts could not be retrieved; 66 unique records were screened. Of these, one was excluded for being a systematic review, and 22 were excluded for not fitting inclusion criteria. Ultimately, 43 studies were included in the qualitative synthesis (see Figure 1). Supplementary Appendix II provides detailed reasons for exclusion of the 20 studies excluded after full text review.

### 3.2 Characteristics of included sources

#### 3.2.1 Overview

This scoping review identified 43 studies employing state-transition and simulation-based modeling frameworks for childhood dental caries. Citation, Country, Study Design, Participants, Context, Concept, and other relevant details can be found in Supplementary Appendix IV. The full extraction table can be found in the Supplementary Table S3. The included studies span four decades (1966–2025) and represent diverse geographic contexts, modeling approaches, and analytic purposes. This section provides an overview of key characteristics that support the inclusion of each study in the review.

**Figure 2:**
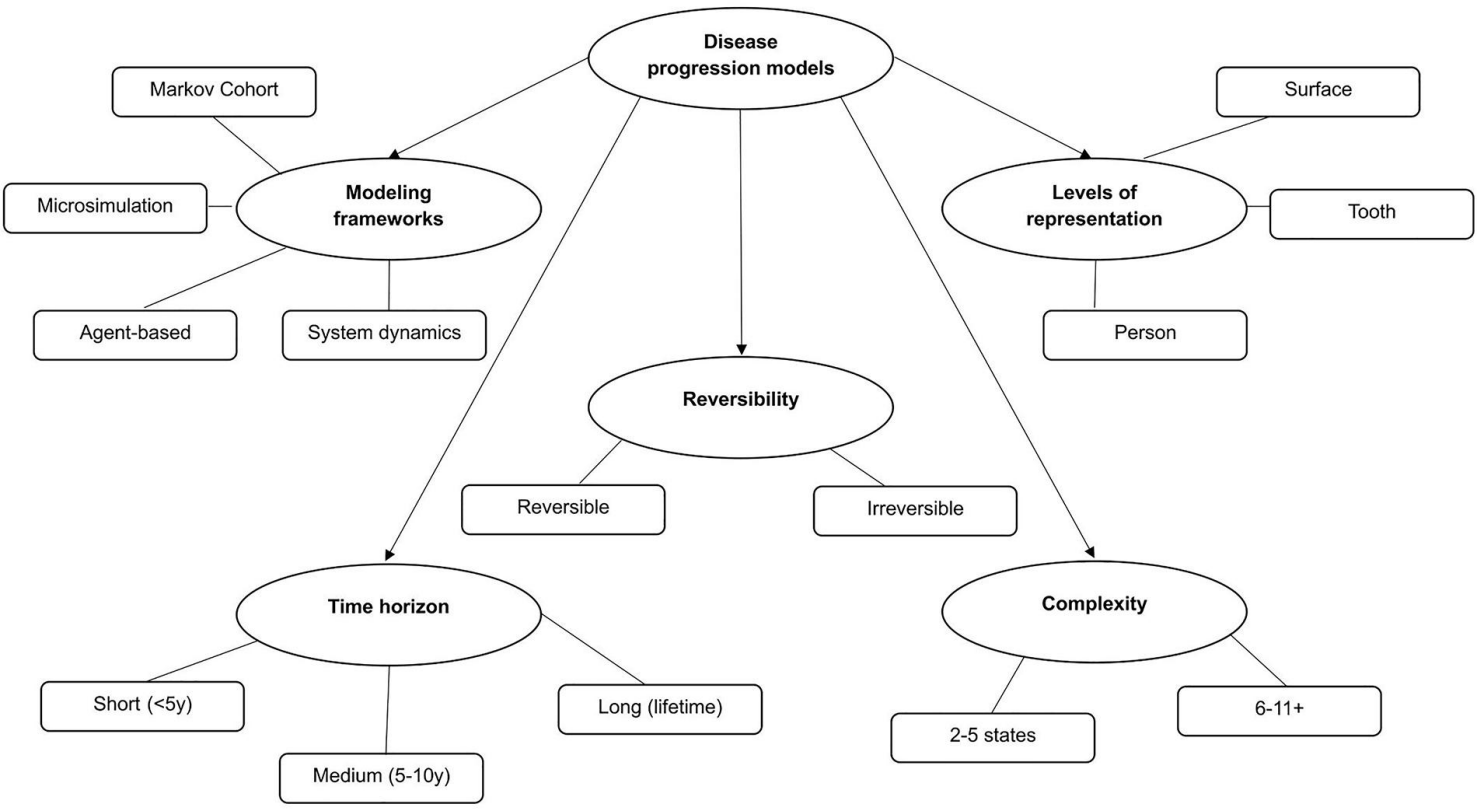
Conceptual taxonomy of caries progression models in children. Alt text: Figure X presents a conceptual taxonomy of modeling approaches, summarizing key dimensions included in the analyzed study.

These studies collectively demonstrate the breadth of modeling approaches applied to pediatric dental caries, reflecting variation in model complexity, temporal resolution, and analytic purpose. To support interpretation of this methodological diversity, Figure 2 presents a conceptual taxonomy of modeling approaches used in pediatric caries research.

#### 3.2.2 Geographic and temporal distribution

The included studies represent international evidence from 13 countries across several continents. The largest contributions came from the United States (*n* = 14 studies), Australia (*n* = 8), and Germany (*n* = 5). Other well-represented countries included Chile (*n* = 3), China (*n* = 2), Canada (*n* = 2), the United Kingdom (*n* = 2), and Belgium (*n* = 2). Single studies were identified from the Netherlands, India, Turkey, South Africa, and Sweden.

Temporally, the evidence base reflects the evolution of modeling methodology in dental health economics. Two foundational theoretical studies from the 1960s established early applications of finite absorbing Markov chains at the tooth-surface level. A sustained increase in published modeling studies occurred from 2005 onward, with all other 41 included studies published in the past two decades (2005–2025). The most recent period (2020–2025) accounts for 17 studies (39.53%), reflecting growing methodological sophistication and policy interest in childhood caries prevention and management.

#### 3.2.3 Study design and modeling frameworks

All included studies employed quantitative modeling approaches using state-transition or simulation-based methods. The predominant framework was Markov-based modeling, used in 35 studies (81.40%) across various implementations: standard Markov cohort models (*n* = 16), Markov microsimulation models (*n* = 11), and decision tree combined with Markov hybrid models (*n* = 8). Additional modeling approaches included statistical estimation frameworks employing Bayesian MCMC and frailty-based multistate models (*n* = 3), Hidden Markov Models designed to correct for misclassification in longitudinal data (*n* = 2), time-dependent non-homogeneous Markov models (*n* = 1), and classical finite absorbing Markov chains with surface-level states (*n* = 2).

To synthesize how modeling approaches relate to disease-state structures and analytic objectives, Table 1 presents a conceptual matrix linking modeling frameworks with the types of disease states typically represented and the analytic lenses applied in the literature. This overview highlights how different modeling approaches tend to align with preventive, restorative, or economic policy analyses.

**Table 1:** Conceptual mapping of modeling frameworks, disease state granularity, and analytic lenses in pediatric caries models.

Modeling framework	Typical disease state structures	Preventive lens	Restorative lens	Economic/policy lens
Cohort Markov models	Simple or severity-differentiated states (e.g., sound → carious → treated/extracted)	✓ Frequently used	△ Limited representation	✓ Common in cost-effectiveness analyses
Microsimulation models	Detailed multi-state structures and restorative cascades	✓ Captures heterogeneous prevention effects	✓ Strong representation of treatment sequences	✓ Enables lifetime economic evaluation
Agent-based models	Patient-level states linked to behavioral or environmental interactions	✓ Useful for behavioral interventions	△ Rarely used	✓ Policy and service utilization modeling
System dynamics models	Aggregated population-level disease states	✓ Population prevention policies	△ Limited clinical detail	✓ Workforce and system-level policy analysis
Surface- or tooth-level multi-state models	Highly granular lesion progression and restoration pathways	✓ Diagnostic or minimally invasive interventions	✓ Captures restorative cycles	△ Used when linked to economic parameters

✓ Commonly used; △ Occasionally used/limited application.

Studies were classified by their primary modeling approach to ensure mutually exclusive categorization. For example, studies described as “Markov model analyzed via microsimulation” were classified as microsimulation when individual-level heterogeneity was central to the analysis, or as cohort models when Monte Carlo simulation was employed solely for probabilistic sensitivity analysis.

#### 3.2.4 Population characteristics and model scope

The included studies modeled diverse population groups with varying age ranges and risk profiles. Studies can be categorized into three groups based on their cohort structure:

Children-only models (in this case, with ages ranging up to 21 years) (69.77%) focused exclusively on pediatric populations. These included early childhood caries prevention studies targeting children aged 0–6 years and school-age intervention evaluations for children aged 6–19 years.

Models that were not limited to children at baseline (*n* = 5) simulated cohorts with age distributions representative of general dental practice populations, including both children and adults. These studies captured the full spectrum of patients seeking dental care in private practice settings.

Risk stratification was explicitly modeled in several studies, with analyses comparing outcomes across low-, medium-, and high-caries-risk populations. Other studies focused specifically on high-risk or vulnerable populations, including children from low socioeconomic backgrounds, Indigenous communities, publicly insured children, and populations with high caries prevalence exceeding 60%.

#### 3.2.5 Time horizons and cycle lengths

Time horizons varied substantially across studies. While some studies examined transitions within short horizons of 1–2 years, most models simulated disease progression over periods of up to 10 years. Twelve studies (18.60%) implemented or included long-term models beginning in childhood and followed pediatric cohorts—starting at ages 6, 8, 12, or 15—through the remainder of the lifespan, with time horizons extending approximately 60–75 years. These models aimed to capture the long-term clinical and economic consequences of childhood caries management decisions, including subsequent restorations, endodontic treatments, extractions, and tooth replacements into late adulthood. All long-term models used cycle lengths of either 6 months (*n* = 7) or 12 months (*n* = 5).

Similar cycle lengths were used in most other models that specified a cycle length. Five studies used alternative cycle structures. Four models applied shorter cycles (1 month, *n* = 3; 3 months, *n* = 1) with time horizons ranging from 2 to 10 years. The objectives of these studies differed: three evaluated the cost-effectiveness of interventions, including two analyses of sealant programs, while one modeled the impact of the COVID-19 pandemic on caries prevalence and disease burden. The remaining study assessed the incremental cost–utility ratio of school-based sealant programs over a 6-year time horizon using 2-year cycles.

Twelve studies focused on time horizons of up to 5 years. Most of these evaluated both costs and outcomes, while a smaller number focused exclusively on intervention effects or disease progression. These shorter horizons primarily represented near-term disease progression and short-term intervention impacts.

Studies with time horizons greater than 5 and up to 10 years showed a similar distribution of objectives. Such medium-length horizons enabled the investigation of intervention outcomes over multiple years as well as mid-term disease progression and economic outcomes over program-relevant periods.

No studies were identified with time horizons longer than 10 years but shorter than 60 years.

#### 3.2.6 Model structure and health states

The structural complexity of included models varied systematically with study objectives. Health state definitions ranged from simple binary classifications to highly detailed restorative cascades.

Table 2 maps disease states across studies, highlighting overlaps and gaps. Mapping disease states across the included models reveals several consistent patterns. Most models incorporate a core set of states representing healthy teeth, carious lesions, restorative treatment, and tooth loss, forming the basic progression structure across studies. However, substantial divergence exists in how disease severity and treatment pathways are represented. Some models distinguish early and advanced lesions using clinical thresholds such as enamel vs. dentin involvement, while others focus primarily on restorative cascades describing sequences of fillings, crowns, and extractions. Preventive models more frequently introduce states such as sealed teeth or arrested lesions, although explicit representation of lesion arrest remains uncommon in economic evaluations. Several potentially relevant state types were rarely represented across studies, including patient-level health states, behavioral states influencing disease risk, and standardized definitions of lesion arrest or remineralization.

**Table 2:** Synthesis of disease state structures used in caries progression models across the included studies.

Model structure type	Typical health states represented	Key overlaps	Notable gaps
Simple models (2–3 states)	Sound/healthy; carious/decayed; sometimes treated or ECC	Universal “Healthy” and “Carious” states	No distinction between enamel and dentin lesions; active vs. arrested states rarely represented
Severity-differentiated models	Normal; early caries (enamel/non-cavitated); advanced caries (dentin/cavitated)	Use of clinical diagnostic thresholds	Restorative outcomes often not included
Prevention-focused models	Sound; sealed (retention/loss); carious; arrested/inactive lesions	Explicit representation of preventive success	Arrested lesions rarely used in economic evaluations
Restorative cascade models (6–11 states)	Sound; decay; filled; refilled; root canal treatment; crown; extraction; bridge; implant; death	Representation of long-term restorative pathways	High data requirements; often assume constant transition risks
Pathology-specific models	Pulp exposure; pulp capping; root-filled tooth; extraction; death	Focus on endodontic disease progression	Rarely used in broader cost-effectiveness models
Surface-level models	Binary states applied to multiple tooth surfaces producing many state combinations	Highest spatial granularity	Computational complexity limits use in economic models

Differences in the number of health states reflect model granularity and are indicative of whether studies focused on basic prevention, detailed treatment pathways, or long-term policy scenarios.

Simple binary or three-state models (*n* = 12) were predominantly used to represent basic health outcomes such as “caries-free” vs. “caries,” sometimes adding a third state for arrested caries, treated teeth, or preventive interventions. These models were typically applied in studies evaluating school-based sealant programs, fluoride varnish application, or home-visit interventions in low- or high-risk populations. One study explicitly noted that “no distinction was made between enamel caries, dentin caries, or pulp involvement” (22) to maintain model tractability.

Four- and five-state models (*n* = 6) incorporated additional treatment or absorbing states, capturing sequences such as sound, decayed, restored, endodontically treated, and missing teeth. These models were commonly used to assess cost-effectiveness of combined preventive and restorative strategies, including targeted professional interventions, resin- or glass ionomer-based sealants, and pulp therapy.

Multi-state models (>5 states; *n* = 9) represented more granular disease progression, distinguishing enamel and dentin lesions, restorations, crowns, bridges, implants, and extractions. These models were frequently employed in long-term economic evaluations, school-based fissure sealant programs, and policy simulations, such as sugar-sweetened beverage taxation or expansion of the dental workforce, where multiple intervention options and tooth-level progression were considered.

Surface-level models (*n* = 9) modeled caries independently for individual tooth surfaces, capturing up to 32 possible health states and enabling detailed analysis of lesion progression, restoration sequences, and preventive effects. Such granularity supported studies evaluating detection technologies, minimally invasive treatments, or surface-specific interventions.

Special or atypical models (*n* = 7) included those combining dental and non-dental outcomes, modeling terminal states (e.g., post-crown), or incorporating advanced statistical techniques to address misclassification, overdispersion, and clustered data. These models were typically used to estimate caries incidence, transition probabilities, and intervention effects in complex or longitudinal datasets.

#### 3.2.7 Intervention types and model applications

The included studies evaluated a diverse range of intervention types, illustrating how modeling objectives drove structural choices. The total number of interventions evaluated or modeled across the included sources was 122, counting each intervention every time it appeared. These interventions were categorized into four groups: preventive, diagnostic/detection, restorative/minimally invasive, and long-term policy/workforce measures. Simpler preventive interventions were typically modeled using fewer health states and shorter time horizons, whereas restorative or policy interventions required more complex structures and longer projections to capture downstream outcomes.

Preventive interventions comprised 32.79% of interventions (*n* = 40) and made up the largest group. These included chemical, physical, application-based, or behavioral measures designed to prevent caries. Specific examples were fluoride varnish (FV), fissure sealants (PFS), probiotics (PB), APF gel, topical fluoride solutions, supervised tooth brushing, oral hygiene instructions (e.g., delivered through home visits or telephone contacts), and broader non-invasive or comprehensive preventive programs.

Diagnostic and detection technologies accounted for 9 interventions. These were primarily aimed at identifying existing or early-stage lesions and included near-infrared light transillumination (NILT), radiographic caries detection (RA/Bitewing), visual-tactile detection (VT/standard of care), screening procedures, and laser fluorescence (LF).

Restorative and minimally invasive pathways represented 36 interventions. These interventions were applied to existing lesions and included atraumatic restorative treatment (ART), root canal treatment (RCT/RoCT), extractions, restorative procedures (fillings, crowns, implants), glass hybrid (GH) or composite (CO) restorations, surgical extractions, direct pulp capping, and chemomechanical caries removal (CMCRA).

Finally, long-term policy assessment and workforce planning encompassed 37 of 122 interventions (30.33%), representing the other large group. These interventions addressed structural, fiscal, or scenario-based measures and included no intervention/control/status quo, sugar/SSB taxes, increased workforce (NHSC), duration and access scenarios, restricted access to care (pandemic scenarios), and cessation of water fluoridation.

#### 3.2.8 Data sources and parameter estimation

Studies drew upon diverse data sources to populate model parameters, with substantial variation in the quality and origin of evidence.

Clinical effectiveness data was most commonly derived from systematic reviews and meta-analyses, particularly Cochrane Reviews for well-established interventions such as fluoride varnish and pit-and-fissure sealants. Several studies used data from randomized controlled trials conducted by the study authors themselves or from published RCTs. Longitudinal observational cohort studies provided transition probabilities for disease progression in several models.

Transition probabilities were estimated using multiple approaches. Direct empirical probabilities were calculated from clinical datasets, including insurance claims databases, program monitoring data, and intervention trial data. Advanced statistical estimation frameworks were employed in several studies, including parametric Weibull regression models, semiparametric approaches with baseline intensities approximated by splines, and frailty models accounting for intraoral correlation and clustering. Expert elicitation was occasionally used for rare events or parameters with limited published evidence. The diversity of data sources and estimation techniques reflects variation in model reliability and highlights methodological trade-offs between empirical rigor and feasibility.

Epidemiological data on caries prevalence and incidence were sourced from national health surveys, including the US National Health and Nutrition Examination Survey (NHANES), the Australian National Child Oral Health Survey, the Dutch National Food Consumption Survey, Chilean national cross-sectional surveys, and country-specific oral health surveillance systems.

Cost data was obtained from standardized fee schedules and tariffs in countries with regulated pricing (e.g., German public and private fee schedules) national health service schedules, professional association fee surveys, insurance claims data reflecting actual paid amounts, and public procurement or microcosting studies.

Several studies incorporated quality-adjusted life years (QALYs) to quantify the overall health impact of dental caries and related interventions. QALYs combine both length of life and health-related quality of life, with utility scores ranging from 1.0 (perfect health) to 0 (death). In dental disease models, utility weights—sometimes expressed as disutility values for conditions such as caries, tooth abscess, or tooth loss—were assigned to health states, while some studies used Quality-Adjusted Tooth Years (QATYs) to capture tooth-level outcomes.

Within state-transition models, patients move between health states such as “Healthy,” “Decayed,” “Restored,” and “Extracted,” and the time spent in each state is multiplied by its utility score to calculate cumulative QALYs over the model horizon. This allows the long-term impact of disease progression and interventions to be quantified in a single health metric. Cost-effectiveness was assessed using willingness-to-pay (WTP) thresholds, where interventions are considered cost-effective if the incremental cost per QALY gained falls below a specified ceiling value (e.g., AUD$28,033 per QALY in Australia; £30,000 per QALY in the UK). Probabilistic sensitivity analyses and cost-effectiveness acceptability curves (CEACs) further illustrate the probability that interventions are cost-effective across a range of WTP thresholds, accounting for parameter uncertainty.

Utility weights for quality-adjusted life year (QALY) calculations were derived from dedicated surveys using validated instruments, including the Child Health Utility 9D (CHU-9D) questionnaire adapted for parent-proxy reporting in young children, published utility decrements from the literature, and newly developed Quality-Adjusted Tooth Year (QATY) measures derived from patient surveys.

#### 3.2.9 Model validation and quality assessment

The rigor of model validation varied substantially across included studies.

Internal validation methods were nearly universal and included face validity assessments by clinical and economic experts (common across most studies), plausibility checks involving simulation of null or extreme parameter values to ensure logical model behavior, structured parameter variation to test key assumptions, and mathematical verification of transition probability matrices and formula implementation.

External validation against independent datasets was reported in several studies: calibration of model outputs to match observed caries prevalence in national survey data with prediction errors below 5%, validation against 3-year randomized controlled trial outcomes, cross-validation through comparison of model predictions with results from similar published studies, and calibration against previous modeling work.

Statistical validation of parameter estimation procedures was conducted in specialized statistical modeling studies through methods such as extensive Monte Carlo simulation studies to assess finite-sample performance, bias, mean squared error, and coverage rates of estimators, and empirical demonstrations of model identifiability.

Several studies reported limited or no formal validation beyond basic plausibility checks, representing a notable methodological limitation. Studies adhering to international reporting standards explicitly noted compliance with ISPOR-SMDM modeling guidelines or CHEERS 2022 reporting standards.

The variability in validation practices indicates differences in model credibility and underscores the importance of transparency in reporting assumptions and testing outputs.

#### 3.2.10 Uncertainty and sensitivity analysis

Many studies conducted formal uncertainty analysis.

Deterministic sensitivity analysis was performed in a multitude of studies. Based on keywords alone, 24 studies could be identified, while a closer examination of the context revealed 31 studies that included deterministic sensitivity analyses. Analyses included univariate (one-way) sensitivity analysis varying individual parameters across plausible ranges or 95% confidence intervals (universal approach), bivariate (two-way) sensitivity analysis examining interactions between parameter pairs, and tornado diagrams displaying the relative influence of each parameter on model outcomes.

Probabilistic sensitivity analysis (PSA) using Monte Carlo simulation was conducted in 32 studies (69.77%), typically involving 1,000–10,000 iterations with parameters sampled from specified probability distributions: gamma distributions for cost parameters (standard approach across studies), beta distributions for probabilities and utilities (standard approach), and normal or lognormal distributions for effectiveness measures. PSA results were presented through cost-effectiveness planes showing the joint distribution of incremental costs and effects, cost-effectiveness acceptability curves (CEAC) showing the probability that an intervention is cost-effective at varying willingness-to-pay thresholds, and acceptability frontiers identifying the optimal strategy at each threshold.

Scenario analyses tested structural assumptions and alternative modeling approaches, including variation in discount rates (0%, 3%, 3.5%, 5%: tested in nearly all long-term models), alternative time horizons (2-year, 3-year, 10-year, lifetime comparisons), different intervention schedules or frequencies, varying population risk profiles, alternative effectiveness assumptions (3-monthly vs. 6-monthly fluoride application), and worst-case/best-case scenarios.

Threshold analyses identified critical parameter values at which decision conclusions would change, including willingness-to-pay thresholds for cost-effectiveness acceptability, minimum effectiveness levels required for interventions to be cost-effective, and price points at which policy interventions become economically favorable.

The range of sensitivity and scenario analyses demonstrates how studies addressed uncertainty and explored the robustness of their conclusions across alternative structural or parameter assumptions.

#### 3.2.11 Economic perspective and discounting

Studies adopted diverse economic perspectives reflecting their policy context and intended audience.

The included studies applied a variety of perspectives in their economic evaluations. The public or health system perspective was the most frequently reported, with nine studies explicitly focusing on costs to public payers, health providers, or the health system as a whole. The societal perspective, which accounts for both direct and indirect costs as well as health outcomes for the population, was applied in four studies, including analyses of fluoride varnish interventions and sugar-sweetened beverage taxation. Another four studies adopted a mixed public-private payer perspective, commonly in the context of the German healthcare system. Similarly, four studies evaluated costs from a payer or provider perspective, focusing on direct costs to insurers, private dental practitioners, or care providers. Finally, a single study considered the consumer or patient perspective, assessing costs at the individual patient level.

Discounting practices followed international guidelines with most studies applying 3% annual discount rates for both costs and health outcomes, particularly common in North American and European studies. Australian studies typically applied 5% discount rates consistent with national health technology assessment guidelines. Nearly all studies tested alternative discount rates in sensitivity analyses (0%, 3%, 3.5%, 5%) to assess the impact on cost-effectiveness conclusions. Short-term studies with time horizons under 2 years did not apply discounting.

#### 3.2.12 Reported strengths and limitations

Authors explicitly reported strengths and limitations of their modeling approaches.

Commonly reported strengths included use of individual-level microsimulation to capture patient heterogeneity, incorporation of quality-of-life outcomes through QALYs or QATYs enabling comparison with non-dental interventions, lifetime time horizons providing comprehensive assessment of long-term consequences, use of high-quality data sources including validated databases and rigorous clinical trials, country-specific or context-specific data enhancing local applicability and policy relevance, comprehensive sensitivity analyses demonstrating robustness of findings (nearly universal), equity analyses stratified by socioeconomic status, and methodological innovations such as first applications in specific countries or populations.

Commonly reported limitations included reliance on low-to-moderate quality evidence for key parameters due to limited research, use of cross-sectional epidemiological data for longitudinal parameter estimation, simplified model structures such as modeling only one tooth or surface per patient rather than full dentition, assumption of constant transition probabilities over time ignoring potential changes in risk, technology, or costs over decades-long time horizons, Markov memoryless property assuming future transitions depend only on current state, not history, assumption of tooth independence ignoring clustering and correlation of caries within individuals, exclusion of indirect costs or broader societal impacts due to chosen perspective, use of utility measures validated in different age groups or cultural contexts than the study population, small sample sizes for utility elicitation surveys, assumption of perfect adherence to intervention protocols, limited follow-up data from short-term trials extrapolated over long time horizons, and lack of comprehensive validation against independent prospective datasets.

Several studies noted specific methodological challenges: For example, Ruff, 2025, noted limitations in capturing intraoral correlation and focusing only on advanced lesions (ICDAS 5/6) (23); Brazzelli et al., emphasized that model conclusions were strongly limited by uncertainty in clinical effectiveness data for HealOzone® (24).

#### 3.2.13 Study quality and methodological rigor

The included studies demonstrated variable methodological quality and rigor. Strengths of the evidence base included widespread adherence to established modeling guidelines in recent publications (ISPOR-SMDM, CHEERS 2022), comprehensive sensitivity analyses in the vast majority of economic evaluations (*n* = 40, 87%), explicit documentation of data sources and assumptions (universal), use of validated data sources including audited insurance databases and national surveys, and increasing adoption of sophisticated methods such as microsimulation and Bayesian approaches in modern studies.

Methodological limitations included heterogeneous validation practices with many studies lacking external validation, reliance on secondary data and literature-derived parameters rather than primary data collection, limited prospective validation of model predictions, inconsistent reporting of uncertainty for all key parameters, and assumptions regarding long-term parameter stability in lifetime models potentially introducing bias.

The specialized statistical modeling studies demonstrated high methodological rigor with formal validation procedures, but these studies focused on parameter estimation rather than full economic evaluation, serving as inputs to inform rather than replace cost-effectiveness analyses.

#### 3.2.14 Rationale for inclusion

Each of the 43 included studies met the eligibility criteria for this scoping review by employing quantitative state-transition or simulation-based modeling methods to evaluate childhood dental caries interventions, outcomes, or policy questions. Studies were included regardless of methodological quality to provide a comprehensive map of modeling approaches used in this field, consistent with scoping review methodology.

The included studies represent the full spectrum of modeling complexity, from simple two-state prevention models to sophisticated lifetime microsimulations with 11 health states and complex restoration cascades. This diversity reflects the evolution of the field and the range of research questions addressed, from short-term program evaluations to long-term policy analysis.

Classical historical studies were included despite their age because they represent foundational theoretical work establishing Markov chain applications in dental epidemiology. Statistical estimation studies were included because they provide essential methodological frameworks for transition probability estimation that inform economic models, even though they do not themselves conduct cost-effectiveness analyses.

The inclusion of both historical and modern studies provides a comprehensive view of methodological evolution and demonstrates how model structure has adapted to different research questions and data contexts.

Complete details can be found in the Supplementary Table S1.

### 3.3 Review findings

Across the 43 included studies, evidence on childhood dental caries modeling reflects a diverse range of interventions, populations, time horizons, and modeling approaches. Preventive interventions—such as fluoride varnish, dental sealants, minimally invasive treatments, and behavioral programs—were most frequently evaluated, often in school- or community-based settings with short- to medium-term follow-up. Diagnostic strategies were less common but emphasized risk-stratified screening and the cost-effectiveness of emerging technologies, including near-infrared light transillumination. Restorative interventions and treatment pathways typically used more complex models to capture long-term outcomes related to restoration longevity, retreatment, and alternative endodontic approaches. Policy and system-level interventions, including sugar-sweetened beverage taxation, water fluoridation, workforce expansion, and national sealant programs, generally employed lifetime horizons to assess broader population health and economic impacts.

Modeling structures varied according to intervention and outcome focus. Preventive and diagnostic interventions often used simpler Markov cohort or microsimulation models with 2–5 health states, whereas restorative and lifetime policy analyses incorporated 6–11 states to account for sequential treatments and long-term consequences. Microsimulation was typically applied when capturing individual heterogeneity, risk stratification, or downstream effects of diagnostic accuracy. Outcome measures ranged from disease-specific endpoints (DMFT/DMFS, prevented lesions) to quality-adjusted outcomes (QALYs, QATYs), with economic evaluations predominating across intervention types.

Several studies explicitly modeled risk-stratified populations, including low-, medium-, and high-risk children and vulnerable groups such as children from low socioeconomic backgrounds or Indigenous populations.

## 4 Discussion

This scoping review mapped and synthesized the current evidence on exclusive, progressive transitionary health states and transition probabilities in children with respect to dental caries. Overall, the included studies demonstrated substantial heterogeneity in their conceptualization of discrete caries states, operational definitions, modeling approaches, and follow-up structures. Despite this variability, most studies employed Markov-type frameworks or other state-transition modeling frameworks, underscoring the continued relevance of such modeling approaches for capturing the dynamic and cumulative nature of caries development in childhood.

### 4.1 Methodological observations

Examining these studies through a methodological lens reveals consistent patterns in how modeling choices reflect—and constrain—the research questions that can be addressed. Across the included literature, broad agreement existed on the directional and progressive characteristics of caries transition pathways, yet only a minority of studies explicitly modeled lesion regression or arrest despite evidence that early lesions can stabilize with preventive care (25). These differences reflect variations in modeling frameworks, from simpler cohort-based Markov models to more detailed microsimulation or surface-level.

The treatment of reversibility in caries state structures varied across models and has important implications for the evaluation of preventive interventions. Many models represented caries as a monotonically progressive process, simplifying model specification and reflecting the clinical reality that cavitated lesions do not heal spontaneously. However, purely progressive models cannot adequately represent interventions targeting early-stage lesions such as fluoride-based therapies or ozone treatment, where observed improvements may be treated as diagnostic misclassification rather than true biological regression.

Population-level variation was infrequently incorporated. Few studies stratified transition probabilities by age, socioeconomic status, or baseline risk factors known to influence caries development. Most studies relied on datasets from specific regions or clinical cohorts, which may limit generalizability to broader child populations.

Most included models operated at the tooth level, reflecting the granularity at which caries develops and data are typically collected. This approach allows precise representation of lesion progression and restorative interventions but introduces challenges when aggregating outcomes to the patient level, since an individual may simultaneously have multiple teeth in different health states. Whether and how aggregation is appropriate depends strongly on the purpose and structural design of the model.

Most included models were structured as closed cohort Markov models, meaning that the modeled population was defined at baseline and no new individuals entered the simulation after it began. Population outflow was typically represented through absorbing states. At the person level, several models incorporated mortality as an absorbing state to account for background death risks over long time horizons. At the tooth level, tooth extraction or tooth loss commonly functioned as the final absorbing state, representing the permanent removal of a tooth from the modeled disease process. In contrast, explicit population inflow—such as births or migration—was generally not modeled. Instead, studies typically initialized simulations with fixed populations, such as hypothetical cohorts of children at specific baseline ages or nationally representative samples followed over defined time horizons. Even in microsimulation approaches that allowed individuals to transition between program participation statuses or risk categories, these transitions occurred within a fixed simulated population rather than an open population structure. This reflects the primary analytic focus of the included models: evaluating disease progression, intervention effects, and long-term outcomes within defined cohorts. Modeling explicit population inflow and outflow is more commonly associated with system dynamics or agent-based models, which are designed to examine population-level service demand or demographic change over time.

Interventions in the included studies were operationalized through several modeling strategies reflecting the study objectives and available data. Mechanistically, interventions acted by modifying transition probabilities between health states (e.g., reducing the likelihood of caries development), applying preventive increments such as reductions in DMFT, or adjusting event risk in stochastic microsimulations. Stratification strategies varied: some studies incorporated age or baseline risk, while others accounted for intra-oral correlation between teeth or focused exclusively on high-risk cohorts. These methodological choices influence interpretation: interventions can be represented at varying levels of detail, with implications for capturing heterogeneity, estimating intervention impact, and linking clinical outcomes to cost-effectiveness. Overall, the operationalization of interventions depended on the purpose of the model, whether evaluating individual-level treatments, cohort-based preventive programs, or population-level policy measures.

Cohort-based Markov models were the most frequently used framework, valued for their conceptual transparency and computational efficiency in health economic evaluation. Their primary limitation is reliance on average transition probabilities, which means that variation in risk profiles, disease histories, or treatment experiences between individuals is not explicitly represented—a meaningful constraint when modeling a disease as heterogeneous as dental caries.

Microsimulation models address this by simulating disease trajectories at the individual level, enabling incorporation of patient-specific characteristics, heterogeneous risk profiles, and complex treatment histories. This greater representational fidelity comes at the cost of substantially higher computational demands and more detailed data requirements, particularly when large numbers of iterations are needed for probabilistic sensitivity analysis.

Agent-based and system dynamics models were not identified among the included studies but represent methodological options relevant to specific research questions. Agent-based models simulate interactions between individuals and their environment, making them suited to behavioral interventions or spatially structured populations. System dynamics models focus on population-level feedback mechanisms, enabling analysis of healthcare service demand or policy effects over time, but at the cost of individual-level detail. The choice among these frameworks depends on the research question, required analytical detail, and data availability (26–28).

### 4.2 Decision-making framework for caries model selection

The methodological diversity identified in this review points to a sequential decision hierarchy for model selection, where each structural choice constrains those that follow.

The first decision is model type, determined jointly by research objective and expected population heterogeneity. Natural history and progression questions are best served by multi-state Markov or semiparametric frailty models, which explicitly represent transitions between distinct disease stages. Economic evaluations favor cohort-based Markov models for their transparency and computational tractability. Population-level policy questions—such as taxation, workforce expansion, or fluoridation—require individual-level microsimulation, since cohort models applying average transition probabilities cannot adequately represent the heterogeneous risk profiles and treatment pathways that determine policy impact. Where substantial population heterogeneity is anticipated, such as in vulnerable or high-risk groups, microsimulation should be preferred regardless of research objective; where the average-patient assumption is defensible, a cohort model suffices.

The second decision is the level of granularity. Surface-level models are appropriate when the intervention acts at the surface, such as diagnostic technologies or minimally invasive treatments requiring high clinical precision. Tooth-level models are suited to questions involving restorative sequences and treatment cascades. Person-level models are necessary when outcomes relate to population health or quality of life. Researchers should resist defaulting to tooth-level modeling simply because data are collected at that granularity—aggregation to the person level introduces assumptions that must be explicitly justified.

Third, model structure and dynamics must be specified, encompassing complexity, biological realism, and time horizon together. Complexity—reflected in the number of health states—should follow from the intervention type: preventive interventions generally require 2–5 states, whereas restorative cascade evaluations require 6–11 or more. The treatment of biological realism is not a stylistic choice: a monotonically progressive, irreversible structure cannot detect the benefit of remineralizing or arresting agents such as fluoride varnish or silver diamine fluoride by design, not by data limitation. When evaluating such interventions, reversible or relapsing-remitting state structures that distinguish between active and arrested lesions are required. Time horizon must equally reflect the dentition type and intervention: short-term horizons of less than five years are generally sufficient for primary teeth and sealant evaluations, whereas permanent dentition models and those examining restorative cycles require lifetime horizons to capture crown failure, endodontic retreatment, and eventual extraction. Compressing the horizon for convenience systematically underestimates intervention value in these contexts.

**Figure 3:**
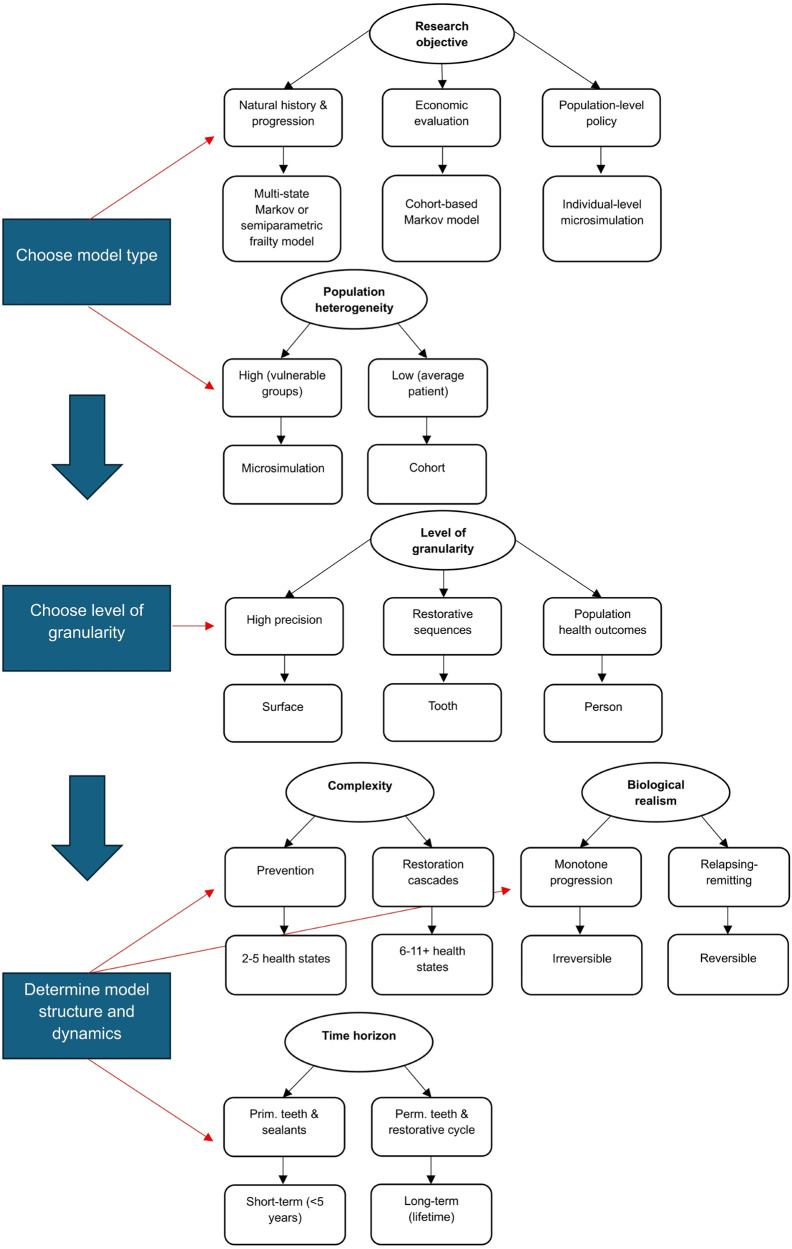
Decision-making framework. Alt text: This figure guides through selecting modeling approaches in dental caries research. The framework integrates research objectives, population characteristics, biological assumptions, and analytical requirements into a sequential decision pathway guiding the choice of model type, structure, and level of detail.

Figure 3 guides researchers through each stage of this decision-making process.

### 4.3 Synthesis and patterns

The literature demonstrates a continuum of methodological complexity. Simple binary or three-state models were predominantly applied for short-term preventive interventions, whereas multi-state and surface-level models were used to simulate long-term restorative or policy-focused interventions. This diversity highlights how modeling approach choices are driven by both the research question and available data, with more granular models supporting evaluations that require individual-level outcomes or nuanced policy simulations.

### 4.4 Limitations of the evidence base

The included studies had several methodological limitations:

- Heterogeneity in study designs, measurement protocols, and follow-up intervals limited direct comparability.
- Health state definitions were not always clearly justified or mutually exclusive, and transition probabilities were sometimes derived from cross-sectional data or short follow-up periods.
- Reporting quality was variable; diagnostic criteria were inconsistently specified, and some studies used older classification systems. For example, when states like “healthy”, “caries”, “extraction”, and “filled” were used instead of the ICDAS.
- Insufficient detail in some sources limited extraction of complete transition matrices.
- Geographic concentration of studies and limited demographic stratification restrict generalizability to diverse populations.

### 4.5 Limitations of the review

This scoping review also has limitations that should be considered when interpreting findings:

- Only English-language publications were included, potentially excluding relevant evidence from other regions.
- Citation searching and backward/forward reference checking were not conducted, which may have missed additional studies.
- No formal critical appraisal of included studies was undertaken, consistent with scoping review methodology, but this limits the ability to evaluate study quality systematically.

Despite these limitations, the review provides a comprehensive mapping of modeling approaches and highlights patterns in model structure, data use, and analytic purpose.

### 4.6 Implications for modeling and decision making

The findings of this review have several implications for the development and interpretation of caries progression models used in research and policy evaluation.

The systematic underuse of reversible state structures across the reviewed literature has a direct consequence for the evidence base: the cost-effectiveness of remineralizing and arresting interventions—such as fluoride varnish and silver diamine fluoride—is likely underestimated in existing models, since monotonically progressive structures cannot capture lesion stabilization by design. This is not a data problem but a structural one, and it means that current economic evidence for non-invasive preventive care in children may systematically understate its value. Future modeling work evaluating such interventions should treat reversible or relapsing-remitting state structures as a methodological requirement rather than an optional refinement.

The fragmentation of granularity choices across the literature—with most models operating at the tooth level despite varying outcome targets—similarly limits the comparability and policy relevance of existing evidence. Tooth-level models are well suited to restorative cascade questions but require explicit and transparent aggregation strategies when person-level outcomes such as quality of life are the ultimate target. The field would benefit from greater consistency in how granularity choices are justified and reported, particularly as quality-adjusted outcomes become more central to pediatric oral health policy.

The concentration of evidence in short- to medium-term horizons, with a gap between 10 and 60 years noted in this review, reflects a broader methodological conservatism that may inadequately serve long-term policy questions. Interventions in the permanent dentition carry consequences extending decades beyond any trial follow-up period, and the restorative cycle—encompassing filling replacement, crown failure, endodontic retreatment, and extraction—can only be captured in lifetime models. Expanding the use of lifetime horizons, informed by non-homogeneous transition probabilities that reflect age-related changes in caries risk, would substantially strengthen the evidence base for childhood caries policy.

Finally, the variability in validation practices identified across studies—with many relying solely on internal plausibility checks—represents a limitation that compounds the uncertainties introduced by structural modeling choices. Decision-makers relying on model outputs for guideline development or resource allocation should prioritize models that explicitly characterize uncertainty, report external validation against epidemiological data, and adhere to established reporting standards (29). Transparent reporting of structural assumptions and parameter sources is essential to ensure that modeling evidence can reliably inform preventive strategies and policy in pediatric oral health.

## 5 Conclusion

This scoping review mapped the existing evidence on exclusive, progressive transitionary health states and transition probabilities for dental caries in children. Interpreted through a methodological lens, the findings reveal that structural modeling choices—including the number of health states, treatment of reversibility, and level of granularity—are the primary determinants of what research questions a given model can credibly address. The review found that transition-based modeling approaches are increasingly used to capture the dynamic nature of caries progression, but the evidence base is highly fragmented. Studies vary widely in how they define health states, apply diagnostic criteria, and report transition probabilities, and few provide empirically derived or mutually exclusive states.

### 5.1 Implications for research

Future research should focus on developing and using standardized, clearly defined health states aligned with established diagnostic criteria. Longitudinal studies with regular follow-up and granular measurement of both progression and regression are needed to generate generalizable transition probabilities. Transparency in modeling assumptions, clear justification of parameter choices, and validation against observed data are essential to strengthen the evidence base. Establishing shared reporting standards for transition-based caries modeling would enhance comparability, reproducibility, and evidence synthesis.

### 5.2 Implications for practice

Although this review did not evaluate intervention effectiveness, consistent and transparent reporting of health states and transitions can indirectly support clinical and policy decision-making. More robust modeling informed by high-quality transition data may improve guideline development, resource allocation, and planning of preventive programs in pediatric oral health.

## Data Availability

The original contributions presented in the study are included in the article/Supplementary Material, further inquiries can be directed to the corresponding author.

## Footnotes

^1^Unless the review developed its own model with the reviewed data.
