# Supplementary material for: State-transition and simulation-based modeling approaches for simulating the progression of dental caries: a scoping review
Source: Front Oral Health. 2026 May 14;7:1791208. doi: 10.3389/froh.2026.1791208 (PMC13216461; doi:10.3389/froh.2026.1791208)
Supplement: Supplementary file 1 [file Datasheet1.docx]

**Supplementary Appendix I**

| MEDLINE (Pubmed)  Search conducted: October 29th, 2025 | | |
| --- | --- | --- |
| Search | Query | Records retrieved |
| #1 | N.A. (see Search Strategy) | / |
| #2 | ("dental caries"[MeSH Terms] OR "dental caries"[tiab] OR "tooth decay"[tiab] OR "caries progression"[tiab] OR "caries lesion*"[tiab]) AND (child[MeSH Terms] OR child*[tiab] OR adolescent*[tiab] OR pediatric*[tiab] OR paediatric*[tiab] OR schoolchild*[tiab]) AND ("Markov"[tiab] OR "microsimulation"[tiab] OR "state transition"[tiab] OR "disease progression model*"[tiab] OR "decision analytic"[tiab] OR "health economic model*") | 53 |
| #3 | #1 AND #2 | 53 |
| Limited to English language. | |  |
| Scopus (Elsevier)  Search conducted: October 29th, 2025 | | |
| Search | Query | Records retrieved |
| #1 | N.A. (see Search Strategy) | / |
| #2 | (TITLE-ABS-KEY("dental caries" OR "tooth decay" OR "caries progression" OR "caries lesion*")) AND (TITLE-ABS-KEY(child* OR adolescent* OR pediatric* OR paediatric* OR schoolchild*)) AND (TITLE-ABS-KEY("Markov" OR "microsimulation" OR "state transition" OR "disease progression model*" OR "decision analytic" OR "health economic model*")) AND (LIMIT-TO(LANGUAGE, "English")). | 67 |
| #3 | #1 AND #2 | 67 |
| Limited to English language. | |  |

**Supplementary Appendix II**

1. Abuduxukuer K, Wang H, Wang C, Luo X, Zeng X, Da D, et al. Prenatal exposure to per-and polyfluoroalkyl substances and its association with Developmental Defects of Enamel (DDE) and dental caries in 4 years old children: Findings from Shanghai birth cohort. Environ Int 2025;198:109411.

Reason for exclusion: Examines associations between PFAS exposure and dental outcomes using regression models; does not model caries progression, health states, or transition probabilities.

2. Afroughi S, Faghihzadeh S, Khaledi MJ, Motlagh MG. Dental caries analysis in 3- 5-years-old children: a spatial modeling. Arch Oral Biol 2010;55(5):374–8.

Reason for exclusion: Uses spatial autologistic regression to assess caries risk patterns; does not model temporal progression or state-to-state transitions.

3. Bandyopadhyay D, Reich BJ, Slate EH. Bayesian modeling of multivariate spatial binary data with applications to dental caries. Stat Med 2009;28(28):3492–508.

Reason for exclusion: Not children, uses Bayesian autologistic regression to model spatial correlation of binary caries outcomes; does not define disease states or model temporal progression.

4. Cho VY, Hsiao JH, Chan AB, Ngo HC, King NM, Anthonappa RP. Understanding Children’s Attention to Dental Caries through Eye-Tracking. Caries Res 2022;56(2):129–37.

Reason for exclusion: Hidden Markov Model applied to eye-tracking data on visual attention; model does not simulate dental caries progression or disease dynamics.

5. Choo-Wosoba H, Gaskins J, Levy S, Datta S. A Bayesian approach for analyzing zero-inflated clustered count data with dispersion. Stat Med 2018;37(5):801–12.

Reason for exclusion: Despite using advanced probabilistic modeling and a longitudinal dataset, it is a statistical inference model, not a simulation or state-transition model describing progressive or exclusive health states over time.

6. Claudia C, Ju X, Mejia G, Jamieson L. The relationship between maternal smoking during pregnancy and parental-reported experience of dental caries in Indigenous Australian children. Community Dent Health 2016;33(4):297–302.

Reason for exclusion: Uses regression to assess associations between maternal smoking and caries prevalence; does not include modeling of disease progression or transition probabilities.

7. da Silva RO, da Silva LB, Pinto Marinho MF, de Souza EER, Cavalcanti YW, Fonseca-Gonçalves A, et al. Cost-efficiency analysis of atraumatic restorative treatment with and without chemical-mechanical caries removal agents based on pain and time for selective removal of carious tissue: a public health perspective. Clin Oral Investig 2025;29(2):134.

Reason for exclusion: No disease progression modeling.

8. Dias S, Welton NJ, Marinho VCC, Salanti G, Higgins JPT, Ades AE. Estimation and Adjustment of Bias in Randomized Evidence by Using Mixed Treatment Comparison Meta-Analysis. J R Stat Soc Ser A Stat Soc 2010;173(3):613–29.

Reason for exclusion: Hierarchical Bayesian model for bias in meta-analysis; does not model caries progression or estimate transitions between disease states.

9. Freitas RD, Moro BLP, Pontes LRA, Maia HCM, Passaro AL, Oliveira RC, et al. The economic impact of two diagnostic strategies in the management of restorations in primary teeth: a health economic analysis plan for a trial-based economic evaluation. Trials 2021;22(1):794.

Reason for exclusion: No actual Markov model results, transition probabilities, or health state simulations are reported yet. The study describes planned methods only, not a completed model.

10. Hu S, Tan SHX, Wang Y, Lai CWM, Gao X, Wee H-L, et al. Cost-Effectiveness of Silver Diamine Fluoride Depends on Caries Activity: A Decision Analytic Model. Caries Res 2025;59(5):425–34.

Reason for exclusion: It’s a decision-tree cost-effectiveness analysis, not a state-transition model (Markov, microsimulation, agent-based, etc.).

11. Karlis D, Ntzoufras I. Bayesian analysis of the differences of count data. Stat Med 2006;25(11):1885–905.

Reason for exclusion: Models correlated paired count data in dental studies; does not define disease states or model temporal caries progression.

12. Manda SOM, Gilthorpe MS, Tu Y-K, Blance A, Mayhew MT. A Bayesian analysis of amalgam restorations in the Royal Air Force using the counting process approach with nested frailty effects. Stat Methods Med Res 2005;14(6):567–78.

Reason for exclusion: Applies Bayesian Cox models to restoration survival; does not define caries health states or model disease progression.

13. Mendoza MAF, Yacapin CPC, Alfaro ACA, Ulitin AR, Valverde HA, Medina VO, et al. Cost-Effectiveness Analysis of Oral Health Care Package of Services within a Comprehensive PhilHealth Benefit Package. Acta Med Philipp 2025;59(14):49–59.

Reason for exclusion: Population-level Markov model for cost-effectiveness of oral health interventions; does not define tooth-level caries health states or model progression in children.

14. Ruff RR, Saxena D, Niederman R. School-based caries prevention and longitudinal trends in untreated decay: an updated analysis with Markov chains. BMC Res Notes 2020;13(1):25.

Reason for exclusion: No explicit modeling of mutually exclusive health states or transition probabilities; study is observational (statistical change in risk, not modeled transitions).

15. Schwendicke F, Stolpe M, Meyer-Lueckel H, Paris S, Dörfer CE. Cost-effectiveness of one- and two-step incomplete and complete excavations. J Dent Res 2013;92(10):880–7.

Reason for exclusion: Uses a tooth-level Markov model where health states are predominantly treatment-based (e.g., restorations, crowns, root canal, implant) rather than pure disease states. The model simulates intervention pathways rather than natural caries progression.

16. Taylor GD, Carr K, Rogers HJ, Vernazza CR. A systematic review of the quality and scope of decision modeling studies in child oral health research. BMC Oral Health 2021;21(1):318.

Reason for exclusion: Systematic Review.

17. Tonmukayakul U, Forrest H, Arrow P. Cost-effectiveness analysis of atraumatic restorative treatment to manage early childhood caries: microsimulation modelling. Aust Dent J 2021;66(S1):S63-S70.

Reason for exclusion: Microsimulation of treatment success, not dental caries progression.

18. Tonmukayakul U, Kularatna S, Atkinson D, Jamieson L, Arrow P. Cost-Utility Analysis of Treatments for Early Childhood Caries in Remote Aboriginal Communities. JDR Clin Trans Res 2025;23800844251346744.

Reason for exclusion: Trial-based cost-utility model for ART-HT; does not define caries health states or model disease progression over time.

19. Ugolini A, Porro F, Carli F, Agostino P, Silvestrini-Biavati A, Riccomagno E. Probabilistic graphical modeling of early childhood caries development. PLoS One 2023;18(10):e0293221.

Reason for exclusion: Although it uses a Markov Random Field (UGM), it models inter-variable dependencies, not disease progression between defined oral health states. No transition probabilities or model structure suitable for health economic modeling.

20. Vanbelle S, Mutsvari T, Declerck D, Lesaffre E. Hierarchical modeling of agreement. Stat Med 2012;31(28):3667–80.

Reason for exclusion: Focuses on statistical agreement indexes (Kappa-like) for examiner ratings; does not model caries progression or estimate transition probabilities between disease states.

21. Wong MCM, Lam KF, Lo ECM. Bayesian analysis of clustered interval-censored data. J Dent Res 2005;84(9):817-21.

Reason for exclusion: Survival model of caries arrest, not simulation of caries progression.

22. Wong MCM, Lam KF, Lo ECM. Analysis of multilevel grouped survival data with time-varying regression coefficients. Stat Med 2011;30(3):250–9.

Reason for exclusion: Uses a survival analysis model to study treatment effects on caries arrest; does not define disease health states or model transitions between caries stages.

23. Yau DTW, Wong MCM, Lam KF, McGrath C. Longitudinal measurement invariance and explanatory IRT models for adolescents’ oral health-related quality of life. Health Qual Life Outcomes 2018;16(1):60.

Reason for exclusion: Study used graded response models for psychometric assessment of oral health–related quality of life; did not model caries progression or simulate disease states.

**Supplementary Appendix III**

|  | [Study title] |
| --- | --- |
| 1a) Author(s), year of publication, country |  |
| 1b) Study design and setting |  |
| 1c) Population characteristics (age range, sample size, risk profile) |  |
| 2a) Type of model (e.g., Markov, microsimulation, agent-based) |  |
| 2b) Model structure and assumptions |  |
| 2c) Health states or disease stages represented |  |
| 2d) Time horizon and cycle length |  |
| 2e) Data sources used to inform the model |  |
| 2f) Validation methods reported |  |
| 3a) Purpose of the model |  |
| 3b) Key outputs |  |
| 4a) Strengths and limitations reported by authors |  |
| 4b) Sensitivity analyses or uncertainty assessments |  |

**Supplementary Appendix IV**

|  | **Citation***  **Country**  **Study design** | **Participants**  **Context** | **Concept** |
| --- | --- | --- | --- |
| 1 | - Bertrand É, Mallis M, Bui NM, Reinharz D (2011), ID-Nr.: 27 - Canada - Cost-effectiveness simulation; Markov model | Cohort of 8-year-old children in Quebec (N=78,372); stratified by low/high caries risk | Three sealant strategies: mixed (current), universal private practice, universal school-based |
| 2 | - Boachie MK, Molete M, Hofman K, Thsehla E (2023), ID-Nr.: 6 - South Africa - Cost-effectiveness analysis; Markov model | Hypothetical cohort 10,000 learners aged 5–15 years; 60% caries prevalence, >80% untreated | Five prevention strategies: ART, fissure sealing, fluoride, sugar reduction, combinations |
| 3 | - Brazzelli M, McKenzie L, Fielding S, Fraser C, Clarkson J, Kilonzo M, et al. (2006), ID Nr.: 10 - UK - Systematic review and economic analysis; Markov decision tree | Hypothetical cohort; fissure and root caries | HealOzone® + current management vs standard management |
| 4 | - Brodén J, Davidson T, Fransson H (2019), ID-Nr.: 33 - Sweden - Cost-effectiveness analysis; Markov simulation | Simulated 1000 permanent posterior teeth; 12-year-old children/adolescents with pulp exposure due to caries | Pulp capping (PC) vs root canal treatment (RCT) |
| 5 | - Chi DL, Van Der Goes DN, Ney JP (2014), ID-Nr.: 16 - USA - Cost-per-event-avoided model; Markov model | First primary molars of Medicaid-enrolled children (10,000 molars simulated); Iowa | Pit and fissure sealants: Standard, Always seal, Never seal |
| 6 | - Choi SE, Shen Y, Wright DR (2023), ID-Nr.: 15 - USA - Cost-effectiveness analysis; Microsimulation | US children 0–19 years (n=10,000 simulated); focus Hispanic/low-income; dental health professional shortage areas | Expanding National Health Service Corps dental workforce capacity (+5–30% funding) |
| 7 | - Choi SE, Simon L (2025), ID-Nr.: 3 - USA - Cost-effectiveness analysis; Stochastic microsimulation | US children 0–19 years (n=8,484 NHANES); publicly/uninsured children disproportionately affected | Impact of stopping public water fluoridation (PWS) |
| 8 | - Choi SE, Wright DR, Bleich SN (2021), ID-Nr.: 26 - USA - Microsimulation model | Children 2–19 years in Supplemental Nutrition Assistance Program (SNAP) (N=10,000 simulated); low-income profile | Restricting sugar-sweetened beverage (SSB) purchases through SNAP benefits |
| 9 | - Curtis B, Warren E, Pollicino C, Evans RW, Schwarz E, Sbaraini A (2011), Id-Nr.: 9 - Australia - Cost-effectiveness analysis based on a cluster-randomized controlled trial; Patient-level microsimulation | 920 patients, representative Australian population age distribution; private dental practice | Caries Management System (CMS) / Monitor Practice Programme vs standard care |
| 10 | - Egil E, Yaylali E (2023), ID-Nr.: 12 - Turkey - Cost-effectiveness analysis; Markov-based decision analytic model | Turkish children 6–15 years; occlusal surfaces first permanent molars | Resin-based vs glass-ionomer sealants vs no treatment |
| 11 | - Espinoza-Espinoza G, Corsini G, Rojas R, Marinõ R, Zaror C (2019), ID-Nr.: 30 - Chile - Cost-utility analysis; Probabilistic Markov microsimulation | 6-year-old children from public schools; low socioeconomic status (SES), high caries risk; first permanent molars (FPM) | School-based "seal all" vs "no seal" program |
| 12 | - Garcia-Zattera MJ, Mutsvari T, Jara A, Declerck D, Lesaffre E (2010), ID-Nr.: 34 - Belgium - Longitudinal analysis estimating prevalence/incidence; Bayesian hidden Markov model | 4468 schoolchildren 7–12 years; four first permanent molars; short-term study | Estimate prevalence/incidence correcting for misclassification and missing data |
| 13 | - Griffin SO, Griffin PM (2016), ID-Nr.: 23 - Australia - Incremental cost-effectiveness analysis; Markov cohort with Monte Carlo | Children 6–48 months; mothers from public maternity clinic, Queensland; high caries risk | Home visits vs telephone intervention vs control for early childhood caries (ECC) prevention |
| 14 | - Griffin SO, Jones K, Crespin M (2014), ID-Nr.: 36 - USA - Methodological model to estimate program impact; Markov model | Children in school-based sealant programs (SBSP); first permanent molars | Develop practical method to estimate prevented caries with minimal dataset (active retention (AR), retention) |
| 15 | - Gupta A, Sharda S, Jyani G, Prinja S, Goyal A, Gauba K (2022), ID-Nr.: 22 - India - Decision-analytic modeling; cost effectiveness analysis | 12-year-old Indian population cohort, followed to age 75 (63-year lifetime) | 20% price increase on sugar/SSBs |
| 16 | - Jevdjevic M, Trescher AL, Rovers M, Listl S (2019), ID-Nr.: 20 - Netherlands - Cost-effectiveness analysis; Tooth-level Markov state-transition | Dutch population 6–79 years (2016 cohort); permanent dentition; societal perspective | 20% ad-valorem SSB tax vs no tax |
| 17 | - Khouja T, Smith KJ (2018), ID-Nr.: 32 - USA - Cost-effectiveness analysis; Markov + decision tree | Hypothetical cohort; occlusal surface first permanent molar (FPM) | Pit and fissure sealant (PFS) vs fluoride varnish (FV) vs no intervention |
| 18 | - Koh R, Pukallus M, Kularatna S, Gordon LG, Barnett AG, Walsh LJ, et al. (2015), ID-Nr.: 5 - Australia - Cost-utility analysis; Markov cohort model | Children 6 months–6 years; disadvantaged, high-risk community, Logan-Beaudesert, Queensland | Home visits vs telephone contacts vs usual care for ECC prevention |
| 19 | - Kopycka-Kȩdzierawski DT, Billings RJ (2006), ID-Nr.: 35 - USA - Statistical model for longitudinal analysis; non-homogenous Markov | Cohort of 631 caries-free children 6–7 years | Time-dependent transitions; influence of salivary mutans streptococci (MS) on caries onset |
| 20 | - Kularatna S, Lalloo R, Kroon J, Tadakamadla SKK, Scuffham PA, Johnson NW (2020), ID-Nr.: 8 - Australia - Cost-effectiveness analysis; Markov model | Indigenous children aged 6–16 years (n=408); remote community, Far North Queensland; >95% Indigenous | Annual professional preventive intervention vs usual care |
| 21 | - Lu KH (1966), ID-Nr.: 38 - USA - Longitudinal analysis; Finite absorbing Markov chains | 5,246 molars (maxillary/mandibular 1st/2nd) from 266 schoolchildren | Actuarial estimation of future dental risks/needs; 32 decay patterns based on 5 surfaces |
| 22 | - Lu KH (1968), ID-Nr.: 37 - USA - Process analysis; Absorbing and ergodotic Markov chains | 184 primary/secondary school children observed 3 years; second upper premolar focus | Caries progression and restoration durability; 5 surface states (healthy, carious, restored) |
| 23 | - Mutsvari T, García-Zattera MJ, Declerck D, Lesaffre E (2012), ID-Nr.: 29 - Belgium - Longitudinal analysis estimating prevalence/incidence; Bayesian hidden Markov model | 4468 schoolchildren 7–12 years; four first permanent molars; short-term study | Estimate prevalence/incidence accounting for misclassification and missing data |
| 24 | - Nguyen TM, Tonmukayakul U, Warren E, Cartwright S, Liew D (2020), ID-Nr.: 39 - Australia - Cost-effectiveness analysis; Markov + decision tree | Individual cohort aged 15+ years, followed to age 85/death; 8 permanent molars; Australian healthcare system | Semi-annual fluoride varnish vs current practice |
| 25 | - Nguyen TM, Tonmukayakul U, Khanh-Dao Le L, Singh A, Lal A, Ananthapavan J, et al. (2023), ID-Nr.: 7 - Australia - Cost-effectiveness analysis | Australian population 2020, ages 0–100 years; stratified by IRSD socioeconomic quintiles | 20% sugar-sweetened beverage (SSB) tax |
| 26 | - Norrie O, Pharand L (2020), ID-Nr.: 4 - Canada - Cost-effectiveness analysis; Markov cohort model | Children aged 1–6 years (n=873) in daycare; low-income communities, Winnipeg | Fluoride varnish program vs usual care (dental general anesthetic) |
| 27 | - Pak D, Li C, Todem D (2019), ID-Nr.: 40 - USA - Semiparametric model development for clustered, interval-censored multi-state data (Markov frailty model) | Low-income children in Detroit (N=1020); 8 primary molars per child; Detroit Dental Health Project (DDHP) | Clustered, interval-censored multi-state modeling with bivariate frailty; intraoral correlation |
| 28 | - Pak D, Li C, Todem D, Sohn W (2017), ID-Nr.: 41 - USA - Statistical model development for correlated, interval-censored longitudinal data (Three-state nonhomogeneous Markov frailty model (Weibull)) | Urban low-income African American children 0–5 years (N=1020); primary molars; DDHP | Correlated, interval-censored data; subject-level frailty; caries progression and spatial symmetry |
| 29 | - Palacio R, Shen J, Vale L, Vernazza CR (2019), ID-Nr.: 24 - Chile - Cost-effectiveness analysis (decision-analytic model: Markov model with decision trees) | Preschool population 4–6 years; low SES; public health perspective | Fluoride varnish (FV) interventions: with/without screening, preschool vs primary care vs counseling |
| 30 | - Pukallus M, Plonka K, Kularatna S, Gordon L, Barnett AG, Walsh L, et al. (2013), ID-Nr.: 42 - Australia - Cost-effectiveness analysis; Markov model | Children 6 months–6 years; mothers from low-SES area, Logan-Beaudesert; public dental service perspective | Telephone-delivered intervention (TI) vs usual care (UC) for ECC |
| 31 | - Quiñonez RB, Downs SM, Shugars D, Christensen J, Vann WF (2005), ID-Nr.: 43 - USA - Cost-effectiveness analysis; Markov + decision tree | Children; occlusal surface first permanent molar; 80% low risk, 20% high risk cohort | Seal All (SA) vs Risk-Based Sealants (RBS) vs Seal None (SN) |
| 32 | - Rodriguez GA, Cabello RA, Borroni CP, Palacio RA (2022), ID-Nr.: 11 - Chile - Cost-effectiveness analysis; Markov decision tree | Preschool children 2.5–6.5 years; low SES | Fluoride varnish (FV) vs probiotic (PB) vs do nothing; with/without school milk program |
| 33 | - Ruff RR (2025), ID-Nr.: 28 - USA - Community-based cluster RCT; Multi-state Markov model (estimation focus) | 1352 children (mean 6.44 yrs); low-income minorities, 29% baseline caries; 6-year molars/premolars | Transition probabilities after silver diamine fluoride (SDF) + fluoride varnish treatment |
| 34 | - Sanghvi R, Cant A, de Almeida Neves A, Hosey MT, Banerjee A, Pennington M (2023), ID-Nr.: 25 - UK - Cost-effectiveness analysis; Markov model | 8-year-old children with 1, 2, or 4 compromised first permanent molars (cFPMs); NHS perspective | Lifetime retention/restoration vs extraction (spontaneous space closure) |
| 35 | - Scherrer C, Naavaal S, Lin M, Griffin SO (2022), ID-Nr.: 17 - USA - Simulation model; Markov chain Monte Carlo | Cohort of 7-year-olds (1000 simulated); low-income households; first molars (1Ms) | Impact of reduced dental care due to COVID-19 on sealants/visits |
| 36 | - Schwendicke F, Basso M, Markovic D, Turkun LS, Miletić I (2021), ID-Nr.: 18 - Multi-national (perspective: Germany) - Long-term cost-effectiveness analysis; Markov microsimulation | 12-year-olds, permanent molars; lifetime follow-up | Glass Hybrid (GH) vs Composite (CO) restorations |
| 37 | - Schwendicke F, Bombeck L (2023), ID-Nr.: 14 - Germany - Model-based cost-effectiveness analysis; Markov microsimulation | 12-year-olds, lifetime follow-up; high-risk group (low SES); posterior permanent teeth | Near-Infrared Light Transillumination (NILT)-based school caries screening vs standard care |
| 38 | - Schwendicke F, Paris S, Stolpe M (2015), ID-Nr.: 2 - Germany - Cost-effectiveness analysis; Markov microsimulation | 12-year-old male, proximal surface; low/high caries risk; mixed public-private payer perspective | Detection-treatment combinations for proximal lesions; radiographic detection with non-/micro-/invasive treatment |
| 39 | - Schwendicke F, Stolpe M, Meyer-Lueckel H, Paris S (2018), ID-Nr.: 13 - Germany - Cost-effectiveness analysis; Markov microsimulation | 6-year-olds, lifetime follow-up; 1000 individuals at low/mid/high caries risk | Fluoride varnish (FV) twice yearly from 6–18 years |
| 40 | - Schwendicke F, Stolpe M, Meyer-Lueckel H, Paris S (2015), ID-Nr.: 1 - Germany - Cost-effectiveness analysis; Markov microsimulation | 12-year-old male, occlusal surface of permanent molar; low/high prevalence populations; German healthcare system | Detection-treatment combinations for occlusal caries; visual-tactile vs radiographic with non-/micro-/invasive treatment |
| 41 | - Warren E, Pollicino C, Curtis B, Evans W, Sbaraini A, Schwarz E (2010), ID-Nr.: 31 - Australia - Cost-effectiveness analysis; Individual-level microsimulation Markov | Individual patient simulation (10,000 patients); representative Australian population age distribution; private practice | Caries Management System (CMS) vs Standard Care (SC); 8 molars modeled |
| 42 | - Xie YY, Cheng ML, Xu MR, Si Y, Xu T (2019), ID-Nr.: 21 - China - Cost-effectiveness analysis; Markov model | 187 children 3–5 years with severe ECC; Beijing; consumer-level perspective | Comprehensive oral health care (education, fluoride, treatment) vs control (exam/counseling) |
| 43 | - Zhou L, Liu B, Li Y, Wang M, Sun C, Zhang X, et al. (2023), ID-Nr.: 19 - China - Multi-perspective multistate Markov Model (CEA/CBA) | 7-year-old children (13.43M nationally); first permanent molars; school setting | Pit and fissure sealant (PFS) application vs no intervention |

*Citation includes author, year, ID number
